# Effect of Chemical Treatment on the Mechanical and Hygroscopic Properties of an Innovative Clay–Sand Composite Reinforced with *Juncus acutus* Fibers

**DOI:** 10.3390/ma18010177

**Published:** 2025-01-03

**Authors:** Hana Ouerghi, Lamine Hassini, Amar Benazzouk, Mohamed Afif Elcafsi

**Affiliations:** 1 Laboratoire d’Energétique et des Transferts Thermique et Massique (LETTM), Faculté des Sciences de Tunis, Université de Tunis El Manar, Campus Universitaire El-Manar, El Manar, Tunis 2092, Tunisia; ouerghihana1994@gmail.com (H.O.); elafif.k@gmail.com (M.A.E.); 2 Applied College—Al Quwaiiyah Branch, Shaqra University, Al Quwaiiyah 19257, Saudi Arabia; 3 Laboratoire des Technologies Innovantes (UR-UPJV 3899), IUT d’Amiens Département Génie-Civil-Avenue des Facultés, Université de Picardie Jules Verne, Le Bailly, CEDEX 01, 80025 Amiens, France; amar.benazzouk@u-picardie.fr

**Keywords:** *Juncus acutus* fibers, alkali treatment, composite, compressive and flexural strengths, capillary water absorption, diffusivity, moisture buffer performances

## Abstract

The viability of using *Juncus acutus* fibers as reinforcement material for developing lightweight sustainable non-structural construction materials in compliance with the valorization of local by-products has been investigated in this work. This study aims to investigate the effect of the chemical treatment of *Juncus acutus* fibers on the mechanical and hygric properties of bio-sourced clay–sand–*Juncus acutus* fiber composite. This lightweight specimen has been produced from a mixture of 60% natural clay and 40% sand by mass, as a matrix, and reinforced with different amounts of Juncus fibers. The fibers were used as a partial replacement of sand in the mixture by volume at 0% (control specimen), 5%, 10%, and 20%. In order to enhance interfacial bonding between the fibers and the binder matrix, which seriously limits the strength development of the composite, the fibers have undergone an NaOH alkali treatment with different concentrations of 1 and 2 wt. %. Morphological and elementary chemical component evaluations based on SEM micrographs and EDX analyses revealed that the 1 wt. % NaOH alkali treatment exhibited the most beneficial effect due to the removal of impurity deposits without significant surface damage to the fibers. This finding was highlighted through the tensile tests carried out which showed the tensile stress value of 81.97 MPa compared to those of the treated fibers with 2% NaOH (74.45 MPa) and the untreated fibers (70.24 MPa). However, mechanical test results, carried out according to the European Standard EN 196-1, highlighted the beneficial effect of the fiber alkali treatment on both the compressive and flexural strengths, particularly for the fiber contents of 5% and 10%, which corresponds to a strengthening rate of 25% and 30%, respectively. The examination of the hygroscopic properties of the samples, including capillary water absorption, water diffusivity, and moisture buffering capacity under the dynamic conditions have indicated that the specimen containing treated fibers exhibited a better moisture regulating property than that obtained with untreated fibers. However, the specimens with treated fibers are classified as excellent hygric regulators based on their moisture buffer values (MBV > 2 g/(m^2^.%RH)), according to the NORDTEST classification. The results also indicated that the capillary water absorption and the apparent moisture diffusivity of composites were lowered due to high fiber-matrix interfacial bond after fiber treatment. Consequently, the composite with treated fibers is less diffusive compared to that with untreated fibers, and thus expected to be more durable in a humid environment.

## 1. Introduction

One of the major sectors that consume a significant amount of total energy in the world is the building sector [1,2]. This leads to a large amount of greenhouse gas emissions [3,4]. To meet this challenge, the academia and industry must develop visionary construction practices to provide innovative lightweight materials that meet the new requirements of users in terms of energy saving, environmental concerns, and also hygrothermal comfort. Bio-based materials have been proposed in recent years as an attractive alternative because they are made from renewable raw materials and allow carbon sequestration thanks to photosynthesis during their growth [5,6,7]. Adding to the environmental benefits, the use of vegetal fibers in construction materials is addressed to provide them some advantages such as low density, insulating thermal capacity, recyclability, low-cost manufacturing, and the ability to regulate indoor humidity buildings [4,8,9,10]. On the other hand, the environmental negative impacts of the conventional building sector resulted in part from the production phase of the binders (Portland cement, hydraulic lime, gypsum, etc.). Indeed, taking the example of Portland cement, the production of this binder is responsible for 5% to 8% of humankind’s CO_2_ gas emissions [11,12].

Therefore, the use of an alternative binder matrix can improve the environmental balance of the composite material while keeping satisfactory mechanical, hygric, and thermal characteristics. In this context, natural clay is promising for low environmental impacts: the resource is available in large quantities; the energy required to extract, transform, and produce materials from earth is extremely low; and it is a recyclable material [13]. As an example, an earth–hemp mixture is estimated to be more than 20 times less costly in gray energy compared with hemp–lime, with a carbon impact of more than 5 times lower [13]. In addition, earth material is highly performant from a hygric point of view due to its capacity to regulate ambient relative humidity [14,15]. Despite these advantages, the use of clay as the sole binder in construction materials exhibited several disadvantages such as low mechanical strength, high water absorption, and durability and shrinkage problems.

Despite the mentioned advantages of the material composite reinforced with natural fibers, the incompatibility of raw fibers with the matrix leads to a significant degradation in the physical, mechanical, and hygric properties of the resulting composites [16,17]. This incompatibility is mainly due to impurities (hydroxyl groups) on the natural fiber’s surfaces, which cause a weakening of the bond strength between the fiber and the matrix [18]. In addition, the use of natural fibers exhibited several disadvantages, such as high water absorption, degradation in alkaline environments, need for alkaline treatments, and heterogeneity, as highlighted in several research works [19].

To overcome this issue, an appropriate physical or chemical pre-treatment of vegetable fibers prior to their insertion in the matrix is required in order to remove the impurities from fiber surfaces and thus enhance the fiber-matrix interfacial bonding [20,21,22]. This leads to a reduction in the volume of voids within the composite, which results in the enhancement of mechanical performances [23,24].

This present research is a continuation of our recently published work [8], which deals with the thermo-mechanical characterization of an innovative clay–sand composite reinforced with *Juncus acutus* fibers. During the elaboration of this lightweight sustainable composite, the Juncus fibers were incorporated into the clay–sand matrix in the natural state without any pre-treatment.

Therefore, the objective of this research is to study the effect of the chemical treatment of *Juncus acutus* fibers on the mechanical and hygric performances of the clay–sand–*Juncus acutus* fiber-based composite. The natural fibers have undergone an alkali treatment with different concentrations of NaOH solution (1 and 2 wt. %), for an immersion duration of 1 h, at laboratory conditions (20 ± 2 °C, 50% RH). The treatment with 1 wt. %. NaOH solution is selected based on the morphological and structural modifications and the tensile stress of the treated fibers. The effect of the selected treatment of fibers on the mechanical and hygric properties of the composite with different amounts of fibers was investigated. The mechanical tests, conducted according to the European Standard EN 196-1, included the compressive and flexural strengths, the corresponding elastic modulus, and the ductility behavior. The hygric properties examined included the water absorption kinetics, the apparent moisture diffusivity, and the moisture buffer capacity (MBV).

## 2. Materials and Experimental Methods

### 2.1. Materials

**Figure 1:**
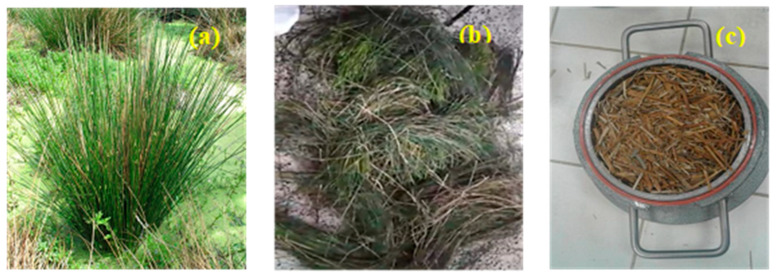
*Juncus acutus* plant (**a**), *Juncus acutus* stems (**b**), and clipped steams called Juncu fibers (**c**).

The Jancus acutus plant (sharp-pointed rush) is a renewable resource, grown spontaneously in abundance in many regions in Tunisia, essentially in the northwest ones. The shape of the natural *Juncus acutus* plant is shown in Figure 1, while the corresponding properties are reported in Table 1. This plant is currently used for a variety of purposes, including as a source of fibers for making paper, baskets, and other woven products, and for medicinal purposes [16,25,26,27]. The natural *Juncus acutus* used in this study was collected from the region of El-Kef city, located in the northwest of Tunisia.

Prior to use as specimen reinforcement, the natural stems measuring 3 to 5 mm in diameter were clipped to the desired length of 1 to 2 cm, and were dried in a drying oven at a temperature of 50 °C. They were then added at the natural state or treated chemically as a partial replacement of sand contained in the control specimen with proportions of 0.6:0.4:0.3 by weight of natural clay, sand, and water, respectively. The level replacements of fibers in the specimen ranged from 0% (control specimen) to 20% by volume.

The sand used in this study is mostly fine-grained according to French standard NF P94-056 [28], with a maximum size of 1 mm. It exhibited 1443 kg/m^3^ in apparent density and 1.32 in fineness modulus. The sand particles were sieved according to the European Standard NF EN 933-2 [29].

Commercially available clay material, already used as a clay plaster inside the house, supplied by “Argilus Industry” which is located in the western region of France, has been used as a binding matrix to produce specimen-based Juncus fibers. Before being used in the mixture, the clay was crushed and sieved with a square mesh of 0.6 mm. The bulk density of the crushed clay is 1380 kg/m^3^ and the Atterberg Limits, including Liquid Limit (W_L_), Plastic Limit (W_P_), and Plasticity index (I_P_) are 20.25%, 17.08%, and 3.17%, respectively.

The chemical composition reported in Table 2 indicated that the used clay consisted essentially of silica, aluminum, and calcium carbonate. In addition, the used clay material also contained significant quantities of carbon (8%) and magnesium (4%) and a small amount of fluorine (0.87%).

The produced specimen consists of clay, sand, water, and treated Juncus fiber mixture, where four sample compositions containing the Juncus fiber content of 0 (control specimen with proportions of 0.6:0.4:0.3 by weight of clay binder, sand, and water, respectively), 5, 10, and 20% vol. were used as natural sand replacement. The composition mixes and the corresponding designation of specimens are reported in Table 3. The physico-chemical properties of the natural fibers and the material composite elaboration procedure have been reported in our previous published works [8].

The elaboration of specimens consisted of first mixing clay and water in a driven mixer, and then the fibers and the sand were gradually added. This process allowed us to overcome the segregation of the fibers in the fresh mix due to their low density, and also to promote their homogeneous dispersion in the mixture. After mixing, the fresh specimen was put into steel molds and then was immediately compacted at a compaction pressure of 0.4 MPa using a static compaction process to ensure the same casting manner for all the specimens. After demolding, the specimens were moist-cured at 20 ± 2 °C and 98% relative humidity condition for approximately 28 days until constant weight. For hardened property measurements, prismatic (70 mm × 70 mm × 280 mm), cylindrical (80 mm × 160 mm), and cubic (70 mm × 70 mm × 40 mm) samples were prepared for mechanical and hygric tests, respectively.

### 2.2. Alkaline Treatment of Natural Fibers

The main objective of the chemical treatment is to roughen the surface of the fibers in order to enhance their adhesion to the binder matrix through the ionization of the hydroxyl group by removing the hydrogen bonding from the fiber structure according to the relationship below [18,30]. In this process, an alkali solution can dissolve a certain amount of waxes, oily contents, lignin, and hemicellulose covering the external surface of the fiber, which increases its roughness [29]. It should be noted that alkaline treatments are very aggressive and can degrade the structure of crystalline cellulose, compromising the strength of the fibers.

Fiber-cell-OH + NaOH ⟶ Fiber cell-O-Na^+^ + H_2_O + impurities

Prior to use as specimen reinforcement, the alkali treatment of Juncus fibers was conducted by immersion in sodium hydroxide (NaOH) solutions, provided by Chimie Plus laboratory (France), at different concentrations of 1 and 2 wt. %, for 1 h, in order to select the better treatment conditions. The companion fibers, taken as reference material, were immersed in water for the same time duration. Afterwards, the alkalized fibers were filtered and washed several times with distilled water to remove any traces of alkali and impurities. The fibers were then dried in an oven at 60 °C for 48 h.

### 2.3. Morphological and Mechanical Characterization of Treated and Untreated Fibers

#### 2.3.1. Morphological and Elementary Chemical Component Characterization

Scanning Electron Microscopy (SEM) was used to examine the influence of chemical treatment on the surface morphology of the *Juncus acutus* fibers. The micrograph analyses were performed using the FEI Quanta 200 Scanning Electron Microscope (USA), conducted at low vacuum scanning conditions, with an accelerating voltage ranging from 5 to 10 kV. The SEM analyzer system was coupled with Energy-Dispersive X-Ray spectroscopy (EDX) to evaluate the effect of 1 wt. % NaOH alkali treatment fibers on the elementary chemical components of hardened clay matrix compared to the control specimen without fibers.

#### 2.3.2. Fiber Tensile Strength Test

**Figure 2:**
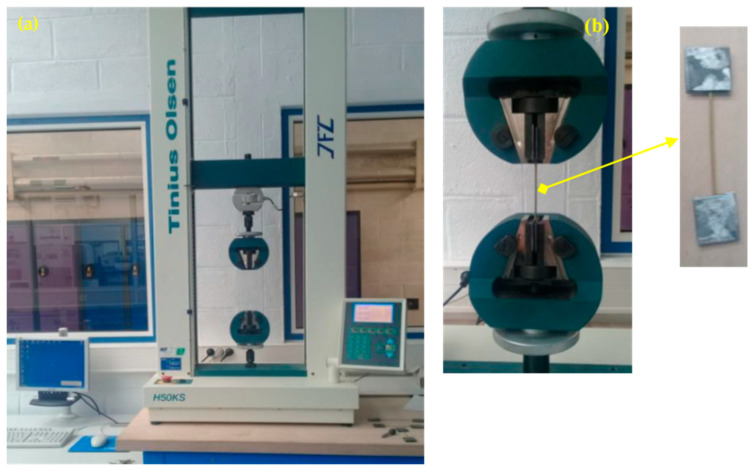
Tensile testing machine (**a**) and the disposition of the *Juncus acutus* stem (**b**).

The tensile strength and elasticity modulus of the treated and untreated Jacutus acutus stems were evaluated by performing a uniaxial tensile test of a single stem according to ASTM D3822 [31]. A universal TINIUS OLSEN H50KS testing machine (Germany) was used under a controlled displacement loading rate of 1 mm/min (Figure 2a).

A total of nine dried *Juncus acutus* stems (treated and untreated), measuring approximately 14 cm in length, were selected for the tensile tests. The ends of each stem were soaked up in epoxy resin and then sandwiched between two aluminum plates of (2 cm × 2 cm) in dimensions (Figure 2b). The aluminum plates fixed on either side of the stem were gripped to the upper and lower hydraulic clamps.

### 2.4. Mechanical Characterization of Material Composite Reinforced with Treated and Untreated Fibers

**Figure 3:**
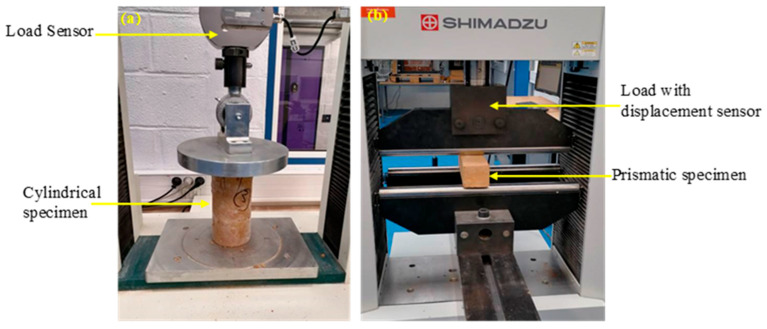
Mechanical test machines: Compressive test (**a**) and three-point bending test (**b**).

The mechanical characteristics of the hardened composite reinforced with different contents of treated and untreated fibers were examined through compressive and three-point bending tests according to the European Standard EN 196-1 [32]. The compressive tests were performed on cylindrical specimens (80 mm × 160 mm) using an electromechanical machine TINUS OLSEN H50KS model (Germany) with a maximum load capacity of 50 KN under a loading rate of 1.6 mm/min (Figure 3a).

Three samples were tested for each composition mix and the average value of the measurement data was reported. The value of the compressive strength σ_c_ and the ultimate strain are given by Equations (1) and (2).

(1) $$\sigma_{c}=\frac{F_{max}}{S}$$

(2) $$\varepsilon =\frac{\Delta l}{l_{0}}$$

where *σ_c_* (MPa) is the compressive strength, *F_max_* (N) is the maximum load, *S* (mm^2^) is the cross-section of the specimen, *ε* (%) is the strain, and ∆*l* (mm) and *l*_0_ (mm) are the displacement and the initial length of the specimen, respectively.

The compressive modulus of elasticity (also called Young’s modulus) was determined from the stress–strain diagram obtained from the compression test. Considering the linear portion of the curve where Hooke’s law is valid, the Young’s modulus *E_c_* (GPa) was calculated using Equation (3).

(3) $$E_{c}=\frac{\sigma }{\varepsilon }$$

The three-point bending tests were performed on the prismatic specimen (70 mm × 70 mm × 280 mm) using a TINUS OLSEN H50KS model testing machine according to the standards EN 196-1 [32]. The tests were conducted with a control deflection rate of 2 mm/min and a span value of 210 mm in length (Figure 3b). The flexural strength values were calculated according to Equation (4).

(4) $$\sigma_{f}=\frac{3}{2}\frac{F\cdot L}{b\cdot d^{2}}$$

where *σ_f_* (MPa) is the bending stress, *F_f_* (N) is the maximum load, and *L*, *b*, and *d* (mm) are the span of the specimen in three-point bending test, the thickness, and the average width, respectively.

The flexural elastic modulus from flexural test was calculated using Equation (5).

(5) $$E_{f}=k\cdot \frac{L^{3}}{4\cdot b\cdot d^{3}}$$

*E_f_* (MPa) is the flexural modulus, and *k* (N/mm) is the elastic stiffness of the specimen, which corresponds to the slope of the linear portion of the load–deflection curve.

### 2.5. Hygric Characterization of the Composite Specimens

#### 2.5.1. Water Transport Property Characterization

**Figure 4:**
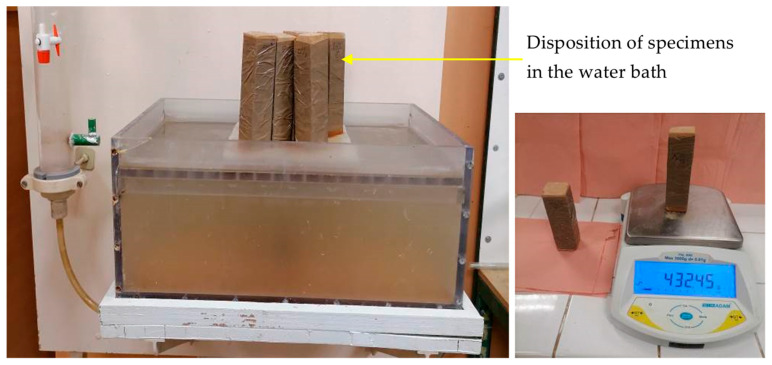
Capillary water absorption setup.

The water absorption tests were performed according to Hall’s work [33]. The test consisted of immersing the base of the dry testing specimens (prismatic specimens with dimensions of 70 mm × 70 mm × 280 mm) in a water bath on a perforated grid at a depth of 3 mm (Figure 4). The wet specimen mass in the function of time was recorded gradually at varied time intervals of 30 s, 1 min, 2 min, 5 min, 10 min, 20 min, 1 h, and then every 2 h through a digital balance placed near the water bath. The water absorption rate (also called the water uptake) was determined using the following Equation (6).

(6) $$M_{t}=\frac{m_{t}-m_{d}}{m_{d}}\times 100$$

where *m_d_* and *m_t_* are the dry sample weight (before initiating the test) and the moist sample weight a time *t*, respectively.

The most important parameter for water absorption is the apparent diffusion coefficient (*D_A_*) because it shows the ability of solvent molecules to penetrate inside the material structure. This coefficient can be calculated according to Fick’s law by using the following Equation (7) [34]:

(7) $$D_{A}=\pi {\left(\frac{h}{4M_{st}}\right)}^{2}.\;{\left(\frac{dM_{t}}{d\sqrt{t}}\right)}^{2}$$

where *M_t_* (%) is the water absorption rate vs. time, given by Equation (6); *M_st_* (%) is the saturation water absorption rate; *D_A_* (m^2^/s) is the apparent moisture diffusivity coefficient; *h* (m) is the height of specimen; and $dMt/d\sqrt{t}$ is the slope of the weight gain versus the square root of time relation (in the linear region).

The side faces of the specimen were waterproofed using a heat-shrinkable plastic film, thus a unidirectional water transfer along the length of the specimen only was assured. This avoids the use of a correction factor in Equation (7) considering the water penetrating through the specimen edges, as used by some authors [33,34].

#### 2.5.2. Moisture Buffer Capacity Evaluation

The study of the hygric behavior of the specimen has been performed by investigating its moisture buffer capacity according to the experimental method addressed by the NORDTEST project [35,36]. The MBV relates the moisture uptake or release per surface area under the cyclic variation in relative humidity, which can be determined from Equation (8).

(8) $$MBV=\frac{∆m}{S\cdot (RH_{high}-RH_{low})}$$

where MBV (g/m^2^.%RH) is moisture buffer value; Δ*m* (g) is the uptake or release of moisture; *A* (m^2^) is the surface area of the specimen; and *RH_high_* and *RH_low_* are high and low relative humidity levels (%).

For the MBV test, specimens measuring 70 × 70 × 40 mm in size were prepared and sealed on the five sides before being stabilized in a desiccator at 50% RH for 48 h. After stabilization, all the samples were placed in a climatic chamber (BiaClimatic type CL2-25-France) and then subjected to cyclic changes in relative humidity with a corresponding duration of 75% RH during 8 h and 33% RH during 16 h at a constant temperature of 23 °C.

## 3. Experimental Results and Discussion

### 3.1. Effect of Alkaline Treatment on Surface Morphology and Tensile Strength of Juncus Fibers

**Figure 5:**
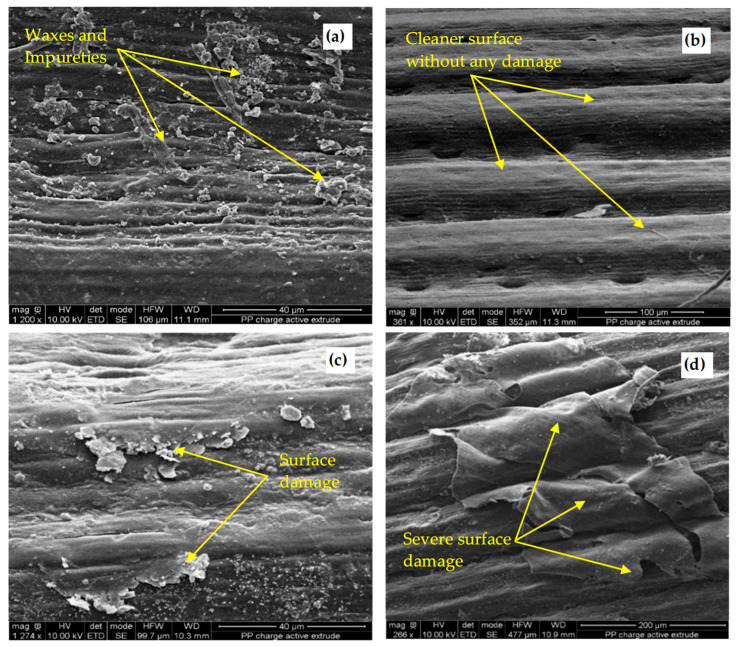
SEM micrographs of natural fiber surface (**a**) and treated fiber surface with NaOH concentrations of 1% (**b**), 2% (**c**), and 10% (**d**).

The fiber surface morphology of a natural fiber and treated fiber with different NaOH concentrations can be analyzed from the SEM images shown in Figure 5. As can be seen, the natural fiber surface is covered by some components that may be the residual of lignin, hemicellulose, pectin, and waxes which are the main reasons for the high sensitivity of the fibers to water absorption, by consequence causing a weak adhesion fiber-matrix [18,37,38]. However, the treatment with 1% NaOH solution cleaned the surface by removing completely the impurities and waxy layers. This phenomenon was observed by many researchers [37,38,39]. It can be observed from the SEM images the appearance of some free spaces between the fibrils that will promote interlocking the bonding between the fiber and the matrix, thus enhancing the mechanical strength of the specimen. For higher alkali concentrations (2%), significant surface damage of the fibers causing their embrittlement due to the severity of the acid attack can be observed [40,41,42].

**Figure 6:**
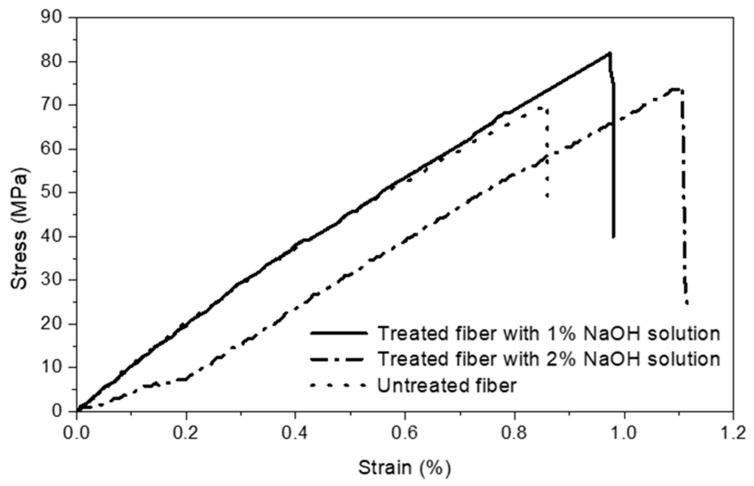
Tensile stress–strain curve of untreated stem and treated stems with 1 and 2 wt. % NaOH alkali solutions.

The average tensile stress–strain curves for the natural and treated *Juncus acutus* single stem with sodium hydroxyl at concentrations of 1% and 2% are depicted in Figure 6, while the correspondent parameter values are listed in Table 4. The results clearly indicated that the stem treated with 1 wt. % NaOH displayed a higher tensile strength (81.96 MPa) than those of untreated (70.25 MPa) and treated with 2 wt. % NaOH (74.45 MPa). The treatment also affects the stiffness of fiber which exhibits an elastic modulus of 5.2% higher and of 13.1% higher in ultimate strain capacity compared to the untreated fiber. The increase in tensile strength, after the treatment process, is possibly due to the removal of non-cellulosic parts and thus increasing the cellulose fractions, which protect the fibrous structure of the fiber against physical and chemical degradations [42]. Table 4, which also compares the mechanical performances of Juncus to Alfa fibers, highlighted the positive effect of the proposed treatment with NaOH on the properties of the studied fibers [4].

### 3.2. Effect of Alkaline Treatment of Fibers on Compressive and Flexural Strengths of Composite

**Figure 7:**
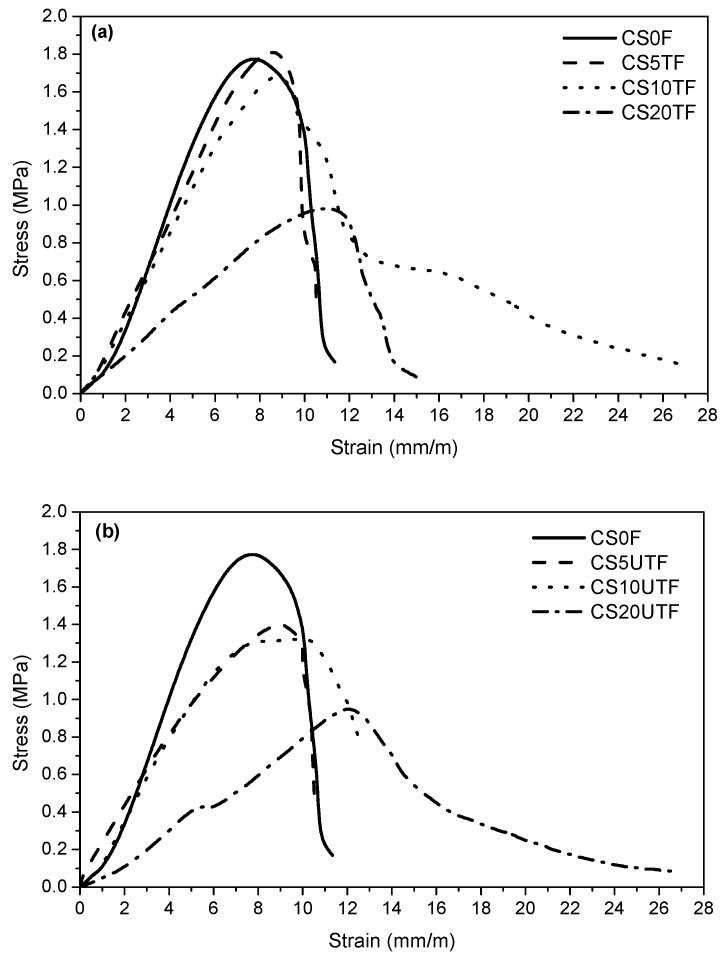
Compressive stress–strain diagrams for specimens reinforced with different volumes of treated fibers (**a**) and untreated fibers (**b**).

The typical stress–strain diagrams of the specimens reinforced with treated and untreated fibers are presented in Figure 7a,b, respectively. The corresponding parameter values, including compressive strength, ultimate strain, and elastic modulus, are reported in Table 5. It can be observed that the two sets of curves exhibit different trends. Indeed, the compressive strength of the specimen reinforced with untreated fibers decreased regularly with increasing fiber volume. However, the specimen reinforced with treated fibers increased to a maximum of 1.81 MPa when the specimen contained 5% fibers, then decreased to a value of 0.94 MPa (remained higher than that of the control specimen) for the specimen with 20% fibers. The comparison of this maximum strength to that of the specimen with the same volume of untreated fibers exhibits a strengthening rate of approximately 25%.

Moreover, the comparison between the compressive strengths of the specimen with treated fibers and that with untreated fibers, averaged over the entire fiber’s levels range, revealed that the alkaline treatment of fibers (by the optimal solution) leads to a significant increase in the average compressive strength from 1.22 to 1.50 MPa. The correspondent strengthening rate is around 20%. This enhancement is attributed to the improvement of the bond between the fibers and the matrix pasta and the stiffness of the fibers, resulting from the removal of impurities and waxy layers covering raw fiber surface. The effect of the alkali treatment of fibers on the improvement of bio-based material compressive parameters was reported by several authors [18,43,44].

According to the results, the treatment of fibers also affects the compressive elastic parameters. Indeed, the average ultimate compressive strain value is dropped from 10.31 mm/m for the specimens with untreated fibers to 9.52 mm/m for those with treated fibers. The average values of the corresponding elastic modulus increased from 141.24 MPa to 178.54 MPa. One can conclude from these last results that for the same volume of fibers, although the specimen with treated fibers has a higher compressive strength, its ductility is decreased compared to that with untreated fibers. This is resulted from a shorter plastic phase and weaker strain capacity before failure due to a reduction in the specific energy absorption. This compressive behavior is explained on one hand by the enhancement of the linking between the treated fibers and the clay–sand matrix, and on the other hand, by the enhancement of the fiber stiffness and the roughness of its surface.

It was interesting to note that during the compressive tests, the control specimen completely disintegrated, while the specimen reinforced with treated fibers maintained its initial structure due to the bridging effect of fibers. This confirmed the ductile behavior for the reinforced specimen and the brittle behavior with sudden fracture for the control specimen. The change in the elastic behavior of the specimens from brittle to ductile has been reported by several authors when vegetable materials were used as additives in concrete based on different binder types [45,46,47].

**Figure 8:**
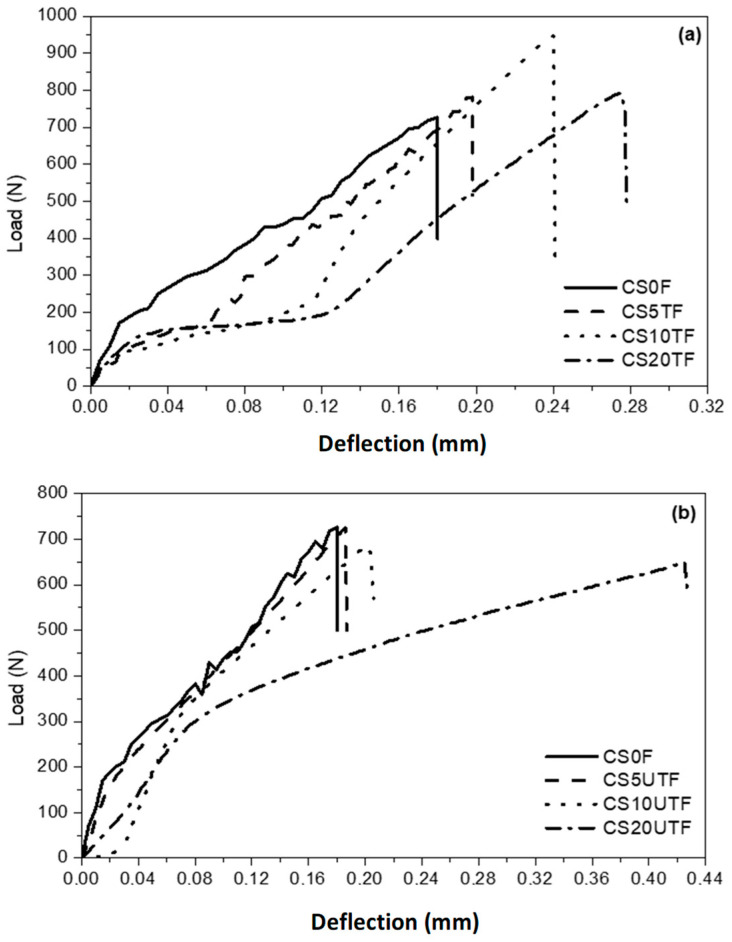
Flexural load–deflection diagrams for specimens reinforced with different volumes of treated fibers (**a**) and untreated fibers (**b**).

The load–deflection diagrams of specimens with reinforced treated untreated fibers are displayed in Figure 8a,b, respectively. The corresponding parameter values including flexural strength, ultimate deflection, elastic stiffness, elastic modulus, and flexural toughness are reported in Table 6. The increase in the untreated fiber volume in the matrix results in a regular degradation of its flexural strength. In contrast, the increase in treated fiber volume in the matrix leads to an improvement in its flexural strength, as compared to the control specimen. Data reveal a maximum strength of 0.87 MPa for reinforced composite with 10% treated fibers. This corresponds to a strengthening rate of about 25% and 30%, as compared to the control specimen and the composite with 10% untreated fibers, respectively. This specific behavior was observed by some authors, such as Zaid et al. [48] when they studied the effect of the treatment of Diss fibers on the physico-mechanical characteristics of Diss concrete based on alternative binder and Ajouguim et al. [43] when they investigated the effect of Alfa fibers on the mechanical and thermal properties of compacted earth bricks.

Based on the results reported in Table 6, the flexural strength, averaged over the entire fiber volume range, increased from 0.62 MPa for the specimens with untreated fibers to 0.77 MPa for that with treated fibers. The corresponding strengthening rate is around 20%. This enhancement could be attributed to the improvement of the links between the fibers and the matrix due to the fiber surface roughness as well as the tensile strength of the fibers resulting in the alkali treatment effect. These findings agree with those obtained by other research works [22,37,47].

The flexural elastic parameters were also affected by the alkali treatment. Indeed, the average ultimate deflection value decreased from 0.81 mm for the specimen with untreated fibers to 0.24 mm for that with untreated fibers. The corresponding elastic modulus increased from 311 MPa to 950 MPa. It should be noted that during the flexural tests, a typical bridging phenomenon was observed for the specimen reinforced with treated fibers, which results in delaying the cracks propagation and retaining the specimen structure.

### 3.3. SEM Micrographs and EDX Analysis

**Figure 9:**
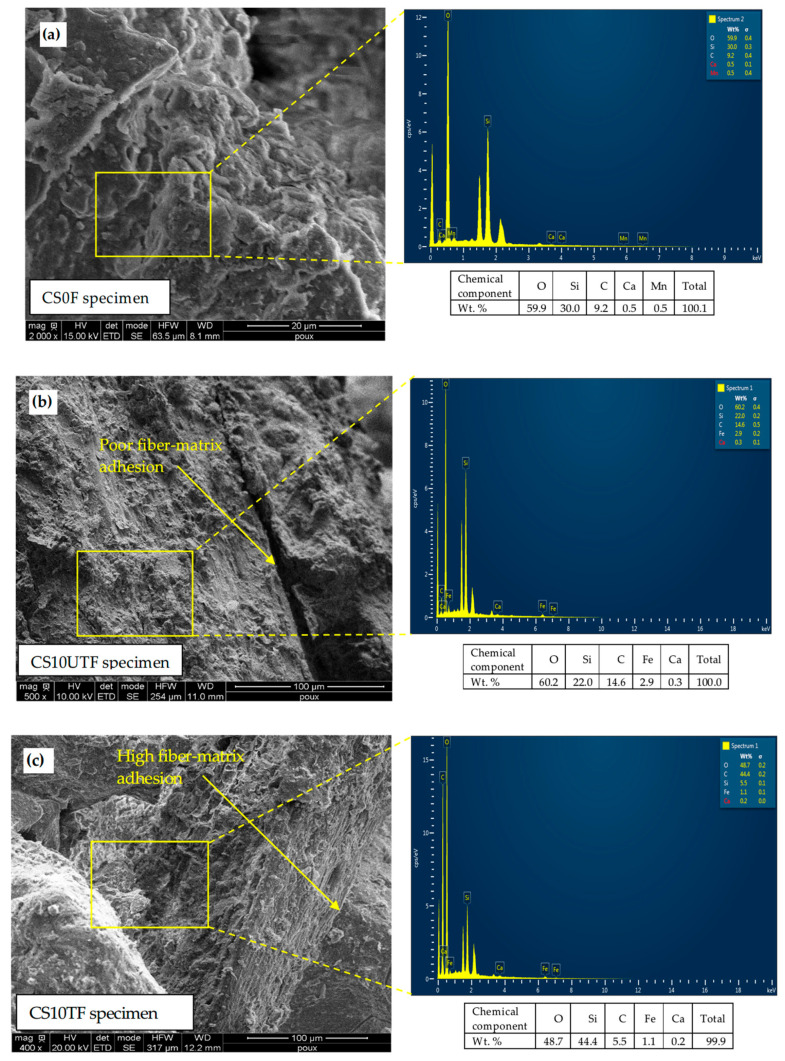
SEM micrographs and X-ray analysis of control specimen (**a**), specimen reinforced with treated fibers CS10F (**b**), and specimen reinforced with untreated fibers CS10TF (**c**).

Figure 9 shows the SEM micrographs and EDX analysis of the fractured CS0F, CS10UTF, and CS10TF specimens. The SEM observations of the CS0F specimen (Figure 9a) display a homogeneous matrix, with high compactness without cracks. The grains of sand are well coated by the clay which resulted in a compact material with higher mechanical strength compared to the CS10UTF and CS10TF specimens. The most dominant elements in the EDX spectra are C, O, Ca, Si, and Fe which are the main components of the clay matrix.

Figure 9b shows the microstructure of the CS10UTF specimen. It can be observed that the addition of untreated fibers leads to poor adhesion between untreated fibers and the matrix. Figure 9c presents the internal microstructure of the CS10TF specimen. It can be shown that the treated fibers are well embedded with the matrix paste, which confirms the treatment efficiency. In addition, the voids were minimized compared to the CS10UTF specimen, and the mortar became denser, which is a major indication of better interfacial adhesion. X-ray analyses in the interfacial transition zone for the CS10TF and CS10UTF specimens reveal that the C, O, and Si elements appear to be the most dominant in the EDX spectra as they are the main components of the lignocellulose fiber structures. Moreover, one can note that the alkali treatment leads particularly to a significant increase in the C element content from 14.6% to 44.4%. This is possibly due to the removal of non-cellulosic parts from the fiber surface and thus increasing the cellulose fractions. In contrast, the Si reduced from 22% to 5.5%, which could be explained by the removal of the rough outer surface of the fiber containing this element after treatment.

It should be noted that the results derived from the SEM observations agree with those reported in other research works focused on the effect of fiber treatment on the morphological characteristics of reinforced composites based on different types of vegetable fibers [49,50,51].

### 3.4. Effect of Alkaline Treatment of Fibers on Water Absorption Capacity and Moisture Diffusivity of the Composite

**Figure 10:**
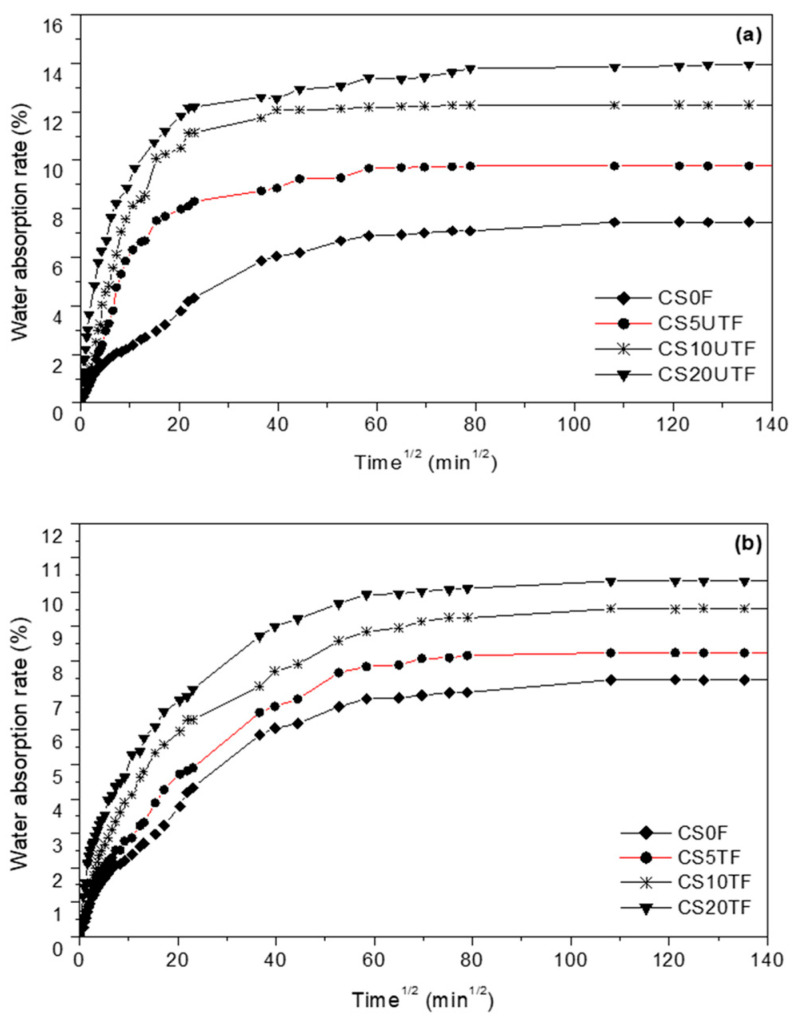
Water absorption rate of specimens reinforced with different volumes of untreated fibers (**a**) and treated fibers (**b**).

The water absorption rate of the specimens reinforced with different levels of untreated and treated fibers as a replacement of sand as a function of the square root of time is given in Figure 10. One can note that water absorption increases with fiber content for the specimens reinforced with treated or with untreated fibers. This behavior was attributed both to the hydrophilic character of fibers and to the segregation of fiber phenomena resulting from fiber overloading. These two factors amplify the porosity that occurs during the drying period of the specimens. This finding agrees with that reported by several authors for bio-based materials [52,53,54]. For all levels of fibers, the water absorption at the saturation point (also called saturation water uptake) for the specimens with the treated fibers is lower than that for the specimens with untreated fibers. The reduction percentage of the water absorption at saturation, averaged over the different levels of fibers, is around 25%. This significant reduction can be explained by the improvement of fiber-matrix interfacial adhesion, which reduced the available polar –OH groups in the system. Moreover, the study showed faster water absorption for the specimens reinforced with untreated fiber. This can be attributed to the large numbers of polar –OH groups, which formed hydrogen bonding between the free –OH groups of the cellulose and water molecules.

For a better understanding of the water absorption mechanisms in the composite reinforced with different levels of treated or untreated fibers, the corresponding moisture diffusion coefficients were estimated based on Equation (6). Table 7 summarizes the obtained results. One can easily observe that the moisture diffusion coefficient for the composite with treated and untreated fibers, increases with increasing the fiber content. However, for a given level of fibers, the moisture diffusion coefficient for composite with treated fibers is lower than that with untreated fibers. This is attributed to the better adhesion between fibers and the matrix. The composite with retreated fibers is expected to be more durable in a humid environment. A similar effect of alkaline treatment was found in several works: Lahouioui et al. [55] for a Date palm fiber-reinforced sand–cement composite and El-Abbassi et al. [54] in the case of Alfa fibers reinforced polypropylene composite.

### 3.5. Effect of Alkaline Treatment of Fibers on Moisture Buffer Value (MBV) of the Reinforced Composite Material

**Figure 11:**
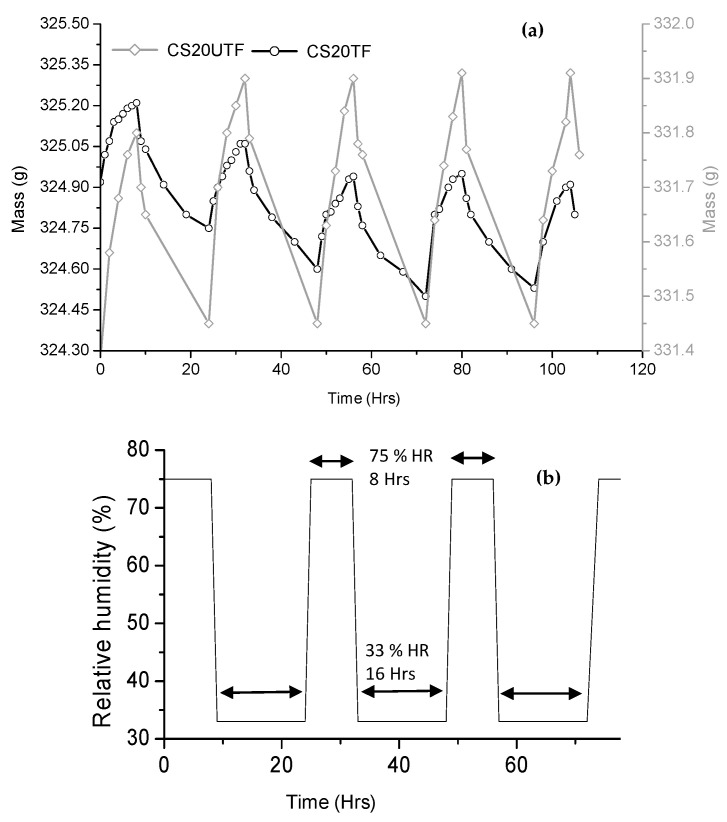
Moisture uptake and release for CS5TF and CS5UTF specimens (**a**), and the cyclic variation in relative humidity in the climatic chamber during the MBV test (**b**).

Figure 11a reported an example of the mass change in CS20TF and CS20UTF specimens, corresponding to the moisture uptake and release when exposed to the RH variations of 8 h at 75% RH level and 16 h at 33% RH level at the constant temperature of 23 °C, as indicated in Figure 11b. Table 8 reported the MBV value for the different formulations (with treated and untreated fibers) as well as their classification according to the Nordtest classification. It can be noted that the MBV values ranged from 2.00 to 2.23 g/(m^2^.%RH) for the specimens reinforced with treated fibers and from 1.82 to 2.18 g/(m^2^.%RH) for the samples with untreated fibers. Consequently, the specimens with treated fibers can be considered as better hygric regulators for hygrothermal comfort than that with untreated fibers. They can be classified as excellent hygric regulators, according to the Nordtest project classification [36].

It should be noted that our results fall generally well within the range of the published ones for composite materials. Indeed, the MBV values range from 1.94 to 2.24 g/(m^2^.%RH) for hemp–lime composites [56], from 1.99 to 2.15 g/(m^2^.%RH) for hemp–concrete composites [57], and from 2.07 to 2.28 g/(m^2^.%RH) for hemp–clay composites [13]. However, the classical masonry materials, like brick and aerated concrete, exhibited significantly lower values of MBV equal to 0.39 and 1.05, respectively [58]. They are classified as limited and moderate hygric regulators, respectively, according to Nordtest classification.

## 4. Conclusions

This study reported the investigation of effective means leading to improving the hygro-mechanical performances of bio-composite material-based clay–sand Juncus fibers through NaOH alkali treatment with different concentrations of 1 and 2 wt. %.

The experimental tests highlighted the following results:

1. Based on the SEM analysis, it was found that the treatment with 1 wt. % of NaOH alkali solution during 1 h is the most effective mean as regards the fiber surface modifications. The treatment will roughen the fiber surface without severe damage, thereby increasing their bonding strength to the binder matrix, which will result in an enhancement of the mechanical strength of the sample. These results are highlighted by the tensile test which shows that the fiber treated with 1 wt. % NaOH displayed the highest tensile strength (81.96 MPa) compared to the untreated fibers (70.25 MPa) and fibers treated with 2 wt. % NaOH (74.45 MPa).
2. The treatment of fibers enhanced the mechanical properties of the resulting composite for the different volumes of fibers. The compressive strength of the composite containing 5% vol. fibers reached a maxima value of 1.81 MPa, while the correspondent flexural strength exhibited a maximum value of 0.87 MPa when the composite contained 10% fibers. This corresponds to a strengthening rate of about 25% and 30%, respectively, when compared to specimens with the same volume of untreated fibers. Otherwise, the composite strengthening rate, averaged over the entire fibers levels range (from 0% to 20%) is about 20% for both the compressive and the flexural strengths. This could be attributed to the enhancement of fiber bonds to the binder matrix resulting from the treatment.
3. Due to the treatment effect, the water absorption at saturation (saturation water uptake) of the composite material, averaged over the different fiber levels, is reduced by approximately 25%. However, the composite water absorption capability is lower for composite reinforced with treated fibers. The treatment of fibers has also a beneficial impact on the composite apparent moisture diffusion coefficient. It results that the composite with treated fibers is expected to be more durable than that with untreated fibers in a humid environment.
4. The MBV values ranged from 2 to 2.23 g/(m^2^.%RH) for composite reinforced with treated fibers and from 1.82 to 2.18 g/(m^2^.%RH) for that reinforced with untreated fibers. Based on these results, formulations with treated fibers are more efficient hygric regulators for hygrothermal comfort compared to those with untreated fibers. Hence, a new sustainable material was proposed based on clay as a binder and *Juncus acutus* as plant fibers. The additional work in the future regarding the bio-composite includes thermal performances and durability under cyclic environmental conditions.

## Figures and Tables

**Table 1:** Properties of used *Juncus acutus* compared to Alfa fibers from Tunisia.

Fiber Type	Bulk Density (kg/m^3^)	Absolute Density (kg/m^3^)	Porosity (%)	Water Absorption (%)
*Juncus acutus*	177 ± 12	1216 ± 25	90.4 ± 4	288.71 ± 12
Alfa [4]	115 ± 10	1549 ± 45	92.6 ± 5	127.0 ± 10

**Table 2:** Chemical composition of used clay.

Oxides	Calcium Oxide	Sodium Oxide	Iron Oxide	Magnesium Oxide	Aluminum Oxide	Silicone Oxide	Total
wt. %	8.39	49.28	0.86	4.39	11.20	25.86	99.98

**Table 3:** Composition mixes of specimens reinforced with different volumes of treated and untreated Juncus fibers.

Specimen Mix Designation	Mass Percentage (%)		Volume Percentage of Fibers, as a Replacement of Sand (%)	Clay (kg/m^3^)	Sand (kg/m^3^)	Fibers (kg/m^3^)	Water/Clay Binder Ratio (by Mass)
	Clay	Sand + Fibers					
CS0F *	60	40	0	733.50	489.00	0.00	0.18
CS5UTF ^1^	60	40	5	733.50	463.53	3.48	0.19
CS5TF ^4^							
CS10UTF ^2^	60	40	10	722.90	432.73	6.87	0.20
CS10TF ^5^							
CS20UF ^3^	60	40	20	688.10	366.21	13.10	0.27
CS20TF ^6^							

* composite specimen with no *Juncus acutus* fibers (control specimen). ^1,2,3^ composite specimen with 5%, 10%, and 20% with alkali untreated fibers, respectively. ^4,5,6^ composite specimen with 5%, 10%, and 20% with treated fibers, respectively.

**Table 4:** Parameter values of treated and untreated *Juncus acutus* under tensile test compared to Alfa fibers.

Fiber Type	Tensile Strength (MPa)	Ultimate Strain (%)	Elastic Modulus (GPa)
Untreated Juncus fibers	70.24	0.82	7.18
1 wt. % NaOH-treated *Juncus acutus* fibers	81.96	0.97	9.56
2 wt. % NaOH-treated *Juncus acutus* fibers	74.45	1.10	6.57
Alfa fibers [4]	94.00	2.02	5.30

**Table 5:** Mechanical properties of specimens reinforced with different volumes of untreated or treated fibers under compressive test.

Specimen Mix	Compressive Strength (MPa)	Ultimate Strain (mm/m)	Elastic Modulus (MPa)
CS0F (Control Specimen)	1.77	7.90	246.4
CS5UTF	1.40	9.052	178.4
CS5TF	1.81	8.702	228.3
CS10UTF	1.32	9.684	168.0
CS10TF	1.68	8.722	208.6
CS20UF	0.95	12.138	77.3
CS20TF	0.98	11.135	98.7

**Table 6:** Mechanical properties of specimens reinforced with different volumes of untreated or treated fibers under flexural test.

Specimen Mix	Flexural Strength (MPa)	Ultimate Deflection (mm)	Elastic Stiffness (N/mm)	Elastic Modulus (MPa)	Flexural Toughness (10^−3^ J)
CS0F	0.66	0.180	4342.7	418.60	48.46
CS5UTF	0.66	0.186	4134.4	398.67	72.23
CS5TF	0.71	0.198	3753.1	361.90	74.31
CS10UTF	0.62	0.201	3667.3	353.62	77.28
CS10TF	0.87	0.244	3486.2	336.17	90.68
CS20UF	0.59	0.425	1846.1	178.00	98.86
CS20TF	0.72	0.275	2606.7	251.36	185.20

**Table 7:** Capillary water absorption properties of specimens reinforced with different volumes of untreated and treated fibers.

Specimen Mix	M_st_ (%)	$dM_{st}/d\sqrt{t}$	D_A_ (10^−6^ m^2^/s)
CS0F	7.45	0.15	2.04
CS5UTF	9.00	0.60	18.95
CS5TF	8.24	0.27	5.39
CS10UTF	12.30	1.00	33.21
CS10TF	9.51	0.40	8.88
CS20UF	13.95	1.60	66.09
CS20TF	10.33	0.80	30.13

**Table 8:** MBV values of the specimens reinforced with different volumes of treated and untreated fibers and their classification according to the Nordtest classification [35].

Specimen Mix	MBV ads. g/(m^2^.%RH)	MBV des. g/(m^2^.%RH)	MBV av. g/(m^2^.%RH)	Nordtest Classification [35]
CS0F	1.70	1.75	1.74	Good hygric regulator
CS5UTF	1.73	1.99	1.82	Good hygric regulator
CS5TF	1.84	1.99	2.00	Excellent hygric regulator
CS10UTF	1.94	1.92	1.91	Good hygric regulator
CS10TF	1.99	2.10	2.04	Excellent hygric regulator
CS20UF	2.10	2.18	2.18	Excellent hygric regulator
CS20TF	1.92	2.19	2.23	Excellent hygric regulator

## Data Availability

The original contributions presented in the study are included in the article, further inquiries can be directed to the corresponding author.
